# Anti-Inflammation of Natural Components from Medicinal Plants at Low Concentrations in Brain via Inhibiting Neutrophil Infiltration after Stroke

**DOI:** 10.1155/2016/9537901

**Published:** 2016-09-05

**Authors:** Jiannan Chen, Xiangjian Zhang, Cong Zhang, Wenhui Wang, Rong Chen, Honglei Jiao, Linlin Li, Lan Zhang, Lili Cui

**Affiliations:** ^1^Department of Neurology, Second Hospital of Hebei Medical University, Shijiazhuang, Hebei 050000, China; ^2^Hebei Collaborative Innovation Center for Cardiocerebrovascular Disease, Shijiazhuang, Hebei 050000, China; ^3^Hebei Vascular Homeostasis Key Laboratory for Neurology, Shijiazhuang, Hebei 050000, China

## Abstract

Inflammation after stroke consists of activation of microglia/astrocytes* in situ* and infiltration of blood-borne leukocytes, resulting in brain damage and neurological deficits. Mounting data demonstrated that most natural components from medicinal plants had anti-inflammatory effects after ischemic stroke through inhibiting activation of resident microglia/astrocytes within ischemic area. However, it is speculated that this classical activity cannot account for the anti-inflammatory function of these natural components in the cerebral parenchyma, where they are detected at very low concentrations due to their poor membrane permeability and slight leakage of BBB. Could these drugs exert anti-inflammatory effects peripherally without being delivered across the BBB? Factually, ameliorating blood-borne neutrophil recruitment in peripheral circulatory system has been proved to reduce ischemic damage and improve outcomes. Thus, it is concluded that if drugs could achieve effective concentrations in the cerebral parenchyma, they can function via crippling resident microglia/astrocytes activation and inhibiting neutrophil infiltration, whereas the latter will be dominating when these drugs localize in the brain at a low concentration. In this review, the availability of some natural components crossing the BBB in stroke will be discussed, and how these drugs lead to improvements in stroke through inhibition of neutrophil rolling, adhesion, and transmigration will be illustrated.

## 1. Introduction

Stroke is the fourth leading cause of death [1]. Of all strokes, subarachnoid hemorrhage stroke and intracerebral hemorrhagic stroke account for 3% and 10%, respectively, while 87% are ischemic stroke [2]. Ischemic stroke starts with rapid reduction of blood flow and oxygen and disorder of glucose and energy metabolism within lesion area, followed by a series of pathophysiology events which are called “ischemic cascades,” including glutamate-induced excitotoxicity, calcium overload, inflammatory response, and cell death [3]. Due to accelerating neurovascular damage and promoting neurorestorative events, inflammatory response plays an extremely important role in ischemic stroke [4]. After cerebral ischemia, inflammation consists of two processes, described as activation of resident microglia/astrocytes and infiltration of peripheral leukocytes [5], resulting in microvessel obstruction, edema formation, cell death, and tissue infarction [6]. For several decades, clinical therapies and basic experiments were focused on neuroprotection, such as anti-inflammation, antioxidative injury, and antiapoptosis [7, 8]. However, the only effective pharmacological intervention to treat stroke is intravascular thrombolysis by administrating recombinant tissue plasminogen activator (r-tPA), which shows therapeutic effects when given within 4.5 hours after onset of symptoms in strictly selected patients. Nevertheless, accompanied with high incidence of intracerebral hemorrhage and narrow therapeutic window, application of r-tPA to clinical patients is limited [9]. Recent studies have revealed that alleviating inflammatory response in ischemic stroke could widen the therapeutic window [10]. Therefore, targeting inflammation could be a crucial and promising therapeutic strategy for preventing brain damage and improving outcomes.

Spencer mentioned that some polyphenols were absorbed to only a limited degree and detected at very low concentrations within the injured brain, so antioxidant activity of these agents failed to account for their neurological improvement ability [11, 12]. But it has become evident that they could protect vulnerable and existing neurons and stimulate neuronal regeneration and neurogenesis to mediate the ability [12]. Similarly, a large number of studies have displayed that anti-inflammatory effects of most natural components on cerebral ischemia have been attributed to their inhibiting resident microglia/astrocytes activation [13–16]. Nevertheless, some drug concentrations in most animal tissues are lower than the concurrent plasma level and the brain level is usually very low or even not detected, because of their poor membrane permeability determined by physicochemical properties [17] and slight leakage of blood-brain barrier (BBB). Therefore, how do they exert anti-inflammatory effects on the brain when they localize in the brain at low or no concentrations? It has been postulated that their anti-inflammatory activities on the brain were mainly mediated by potential to downregulate expression of inflammatory mediators and ameliorate leukocyte infiltration. As a matter of fact, emerging evidence has revealed that they were able to peripherally inhibit leukocyte infiltration at the level of inflammatory mediators. Some natural compounds applied commonly, like sodium *β*-aescin, alleviate neutrophils adhesion to endothelium by downregulating the expression of cellular adhesion molecules (CAMs) [18–20]. This present review will attempt to clarify the probable mechanisms in which certain natural compounds exert such effects on the brain.

**Figure 1:**
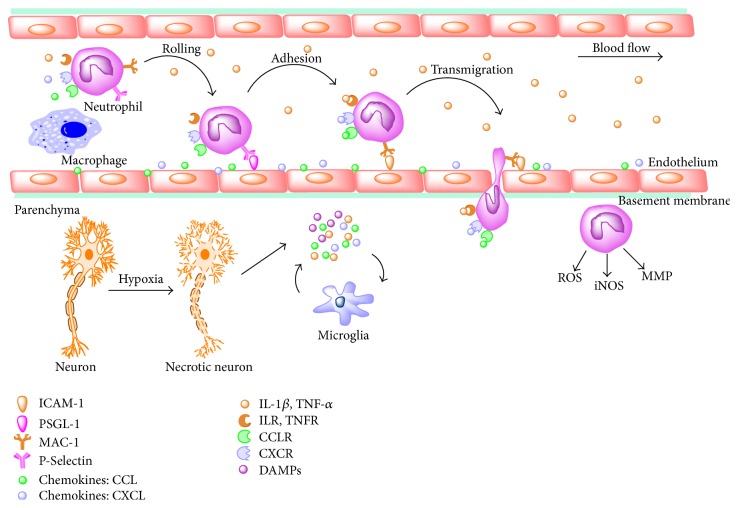
The process of neutrophil infiltration into the cerebral parenchyma. Injured brain cells release DAMPs and cytokines within lesion area and into the vessel lumen. Subsequently, CAMs on the surface of endothelium and neutrophils are induced, participating in the series steps of neutrophil rolling, adhesion, and transmigration. ICAM-1, intercellular cell adhesion molecule-1; PSGL-1, P-selectin glycoprotein ligand 1; MAC-1, macrophage adhesion molecule 1; CCL, monocyte chemoattractant protein; CXCL, chemokine (C-X-C motif) ligand; IL-1*β*, interleukin-1*β*; TNF-*α*, tumor necrosis factor-*α*; ILR, interleukin receptor; TNFR, tumor necrosis factor receptor; CCLR, monocyte chemoattractant protein receptor; CXCR, chemokine (C-X-C motif) receptor; DAMPs, damage-associated molecular patterns.

Data have revealed that an increased number of leukocytes were detected in the microvessels and parenchyma in lesion area [21], mainly in the border zones of infarcts, ischemic penumbra, but not in the normal brain [22]. In the local ischemic brain, leukocyte (especially neutrophil) recruitment cascade is generally subjected to rolling, adhesion, and transmigration [23] (Figure 1). This cascade starts with release of specific inflammatory cytokines, such as tumor necrosis factor-*α* (TNF-*α*), interleukin-1*β* (IL-1*β*), and interleukin-6 (IL-6), inducing the expression of CAMs on endothelium surface [24]. Hence, targeting neutrophil activation and recruitment has been evaluated as stroke remedies [25].

## 2. Impact of Experimental Stroke Models and the BBB Openings on Penetrability of Drugs

Forms of human stroke are various and complicated, and no single animal model can encompass all of the variables on behalf of the events in human ischemic stroke [26]. Reliable animal stroke models provide powerful evidence of efficacy and safety for these drugs to be applied to clinical patients [27]. In order to analyze mechanisms in ischemic stroke, researchers have established less than 10 rodent models of focal cerebral ischemia used in experiment, such as permanent or transient middle cerebral artery (p/t-MCAO) or distal middle cerebral artery occlusion (p/t-dMCAO) by ligating, clipping, photothrombosis, electrocoagulation, or thread [28]. The coagulation model, characterized as distal, permanent MCAO by transcranial electrocoagulation, fulfills criteria of clinical relevance, low mortality, and high reproducibility. Also, the resulting infarct of the model is located mainly in the cortex and the infarct volume corresponds to that of majority of clinical patients [29]. Published study of our laboratory revealed that BBB leakage in t-MCAO mice, given Evans blue (EB) via tail vein injection at 2 h before execution, was measured at 24 h after surgery and the extensive blue area indicating BBB disruption was corresponding to visualized infarct size [30]. Of interest is another data set unpublished in our laboratory, showing that the blue area in p-dMCAO mice, of which EB administration method was the same to that of t-MCAO mice, was not detected obviously owing to the permanent occlusion of the distal MCA and blocking partly EB delivery in the vessel. Therefore, many medicines that cannot pass through the BBB under physiology conditions, even if the brain undergoes ischemic injury, are most likely not to penetrate the BBB efficiently to function at early phase, which at least exists in the p-dMCAO models.

The BBB is formed by microvessel endothelial cells in the central nervous system (CNS) and acts as an interface keeping brain's internal milieu from the rest of the body structurally and functionally, regulating the migration of molecules, ions, and cells strictly [31–33]. It is possible for small molecules with molecular weight < 400 Da and forms < 8 hydrogen bonds to cross the BBB via free diffusion mediated by lipid in principle, while majority of small molecule drugs factually fail to pass, as well as all large-molecule drugs, especially products of biotechnology [34]. However, inflammatory response cripples BBB integrity, described as upregulation of number of endothelial caveolae and rate of transcytosis after t-MCAO, as well as disruption of tight junction (TJ) morphology and enhanced paracellular BBB permeability [35]. Ultimately, some drugs prevented physiologically are allowed to cross the BBB to function under pathological conditions. And there are two openings of BBB after MCAO [36]. The EB extravasation reached two peaks at 6 h and 48 h after cerebral ischemia/reperfusion and decreased at 24 h [37]. Another data set showed that there was a 200-fold increase in barrier permeability to FITC-albumin at 6 h and a further period of disruption around 24 h [38]. Many researches revealed that two openings of BBB permeability appeared at 3–6 h and 48 h or 72 h after ischemia/reperfusion, respectively [39–41]. In fact, changes in TJ permeability following ischemia may not be measured immediately but after continuous hours of reduction in cerebral blood flow, inducing an observable increase in paracellular permeability [42]. Knowland considered that crippling barrier function occurred as early as 6 hours after stroke, and TJs related to paracellular diffusion displayed significant structural defects only after 48 hours [35]. Many drugs with short half-life, administrated only once before the first opening or between the two openings of BBB for anti-inflammation, are absorbed rapidly and metabolized extensively before passing completely through BBB. Thus, single-dose administration of these drugs may not reach the effective concentration to exert anti-inflammation on the ischemic area except given mega doses and repetitive doses.

Equally importantly, the ability of drugs to localize in the brain at an efficient concentration also depends partly on their physicochemical properties, including molecular weight, hydrogen-bonding capacity, and number of rotation bonds (NROTB), of which the favorable values are <500 Da, <12, and <10, respectively. Drugs with poor membrane permeability (more than the favorable value) have poor intestinal absorption, leading to low concentration in most tissues of animals, especially in the brain [17].

## 3. Neutrophils and Inflammatory Mediators in Ischemic Stroke

Inflammation plays an important role in ischemic stroke pathophysiology, particularly in the cerebral ischemia/reperfusion [3]. Inflammatory cells* in situ* including microglia and astrocytes, as well as infiltrated macrophages, and neutrophils from peripheral blood are involved in poststroke inflammatory response [43]. Many studies showed that leukocytes increased in the microvessels and parenchyma of lesion area as early as 30 min after artery occlusion onset [44, 45]. In particular, neutrophils are among the primary cells in the blood responding to ischemia and associated with increased infarct volumes in ischemic stroke [46]. After stroke, necrotic lesion produces abundant damage-associated molecular patterns (DAMPs) [47], such as mitochondrial DNA [48], ATP [49], and carboxyalkylpyrroles [50], to which injured brain cells exposed secret inflammation mediators, such as platelet-activating factor (PAF), TNF-*α* and IL-1*β*, and chemokines [51, 52]. Subsequently, the expression of CAMs on the surface of endothelium is induced [53]. Neutrophil rolling depends mostly on selectins, while adhesion and transmigration are integrin-dependent [23]. Once present on the surface of endothelial cells, these CAMs interact with complementary receptors on neutrophils surface; for example, P-selectin binds to its ligands-P-selectin glycoprotein ligand 1 (PSGL1) [54, 55], resulting in neutrophil rolling on the surface of endothelium along the direction of blood flow [23], and the interactions between neutrophil macrophage adhesion molecule-1 (MAC-1) and endothelial intercellular adhesion molecule-1 (ICAM-1) contribute to neutrophil adhesion to activated endothelium [56]. Consequently, neutrophils transmigrate into the brain parenchyma via orderly adhering to the endothelium and crossing the microvessel wall. Also, macrophages and monocytes enter the ischemic region and promote the inflammatory response predominantly 5 to 7 days after ischemia [53]. Infiltrated neutrophils have a remarkably destructive potential in several ways. Activated neutrophils infiltrating into the parenchyma produce many inflammatory factors including proteases (matrix metalloproteinases and elastase), reactive oxygen species (ROS) [23], and inducible nitric oxide synthase (iNOS) [53], facilitating inflammatory response, BBB breakdown, and cell death, as well as affecting brain repair. Additionally, neutrophils release tissue factor and neutrophil extracellular traps (NETs) acting on coagulation and interact with platelets [57, 58], leading to enhanced platelet aggregation and thrombus formation [59]. Also, influx of neutrophils contributes to neurotoxicity from the release of decondensed DNA [60]. Recently, researchers have focused on targeting neutrophils specifically as cerebroprotective strategies through neutrophil depletion, inhibition of inflammatory mediators, or neutrophil function to reduce infarct size and improve outcomes [21].

Inflammatory mediators, including cytokines, chemokines, and CAMs, play pivotal roles in the process of neutrophil activation, rolling on and adherence to the activated endothelium of blood vessel walls, and transmigration into the cerebral parenchyma. Cytokines, such as TNF-*α* and IL-1*β*, contribute to neutrophils and endothelium activation via upregulating the expression of CAMs [61]. TNF-*α* mRNA elevated in low levels from 30 minutes to 6 hours after occlusion and a rapid increase of IL-1*β* were observed as soon as 1 to 2 hours after occlusion, and they both persisted for approximately 5 days [62]. Modulating the function of TNF-*α* and IL-1*β* has remarkable effects on evolution of the infarct in both experimental and human stroke at the early phase of stroke, corresponding to the therapeutic window (<4.5 h) [4, 62, 63]. Emerging evidence displayed that inflammatory cytokines increased both centrally and systemically after ischemic stroke and correlated with infarct volume and stroke severity, and the systemic cytokine response paralleled the events in the CNS, suggesting that serum TNF-*α* and IL-1*β* had a predictive value for stroke outcome [64, 65]. Intraventricular injection of recombinant IL-1*β* immediately after reperfusion remarkably aggravated brain edema in a dose-dependent manner, increased brain infarction volume, and facilitated neutrophils adherent to the endothelium and infiltrating into ischemic areas, which were reversed by injection of anti-IL-1*β* [66]. Consistently, IL-1*β* deficient mice presented smaller infarct volume [67]. Interestingly, peripheral administration of recombinant human interleukin-1 receptor antagonist significantly inhibited infarct size and cerebral edema formation by 46% and 49%, respectively, after MCAO [68]. In experimental stroke models, administration of neutralizing antibodies to TNF or TNF-binding protein had protective effects [62]. CAMs, mainly including selectins, integrins, and immunoglobulin, expressed on leukocytes and endothelium, contribute to the process of inflammatory cells rolling, adhesion, and transmigration [24, 69]. CAMs (Table 1), especially ICAM-l, P-selectin, and E-selectin, are induced by cytokines after stroke and are responsible for the interaction between leukocytes (particularly neutrophils) and vascular endothelium [61]. In p-MCAO rats, levels of ICAM-1 mRNA in the ischemic cortex increased significantly at 3 h (2.6-fold) and peaked at 6 to 12 h (6.0-fold), while those in t-MCAO rats were 1 h (6.3-fold) and 12 h (12-fold), respectively. And they both remained for 5 days [70]. In experimental stroke models, blocking ICAM-1 reduced infarct volume, decreased mortality, and improved outcomes via pharmaceutical intervention and genetic deletion, as well as immunodepletion of neutrophils volume [71, 72]. P-Selectin and E-selectin on the endothelial cell surface are upregulated in response to ischemia, which promotes neutrophil adhesion by binding PSGL-1 [52, 55]. Expression of E-selectin was upregulated in the ischemic cerebral vasculature within 4 hours of reperfusion and lasted for 24 hours [73]. In rodent ischemic stroke, blocking E-selectin or P-selectin with antibody or by knockout decreased infarct volume and improved functional outcomes [73–75]. Knockout of MAC-1 reduced neutrophil infiltration and infarct size after cerebral ischemia [76]. In rodent and rabbit stroke, treatment with a humanized MAC-1 monoclonal antibody (Hu23F2G) crippled neutrophil migration and reduced ischemic injury [25, 77]. In addition, free radicals generation leads to increased expression of selectins on leukocyte surface and hypoxia can directly stimulate the upregulation of ICAM-1 and neutrophil adherence to endothelium through interaction of MAC-1 with ICAM-1 [4]. Chemokines, lining the luminal part of endothelium, are able to induce changes of integrins on neutrophil surface [23] and guide the migration of blood-derived inflammatory cells towards the source of the chemokines, which are indispensable in inflammatory cells recruitment [61]. The level of chemokines, such as monocyte chemoattractant protein-1 (MCP-1) and macrophage inflammatory protein-1a (MIP-1a), has been found to increase in experimental stroke and low expression of chemokines is associated with reduced injury by inhibition or deficiency [78, 79], attributed to impairing leukocyte infiltration [80]. Data showed that MCP-1 mRNA is increased at 6 hours after MCAO [81] and remained for 5 days [82]. Injection of a chemokine-receptor antagonist into cerebral ventricular displayed a reduction of infarct volume [21]. As mentioned above, crippling neutrophil infiltration peripherally to reduce cerebral ischemic damage and improve neurological deficits can be carried out through several mechanisms including reducing neutrophil activation and recruitment, blocking neutrophil adhesion to endothelium, and transmigration at the level of cytokines, chemokines, and CAMs (receptors and CAMs on neutrophils are illustrated in Figure 4).

## 4. Natural Components from Medicinal Plants Inhibiting Neutrophil Infiltration

**Figure 2:**
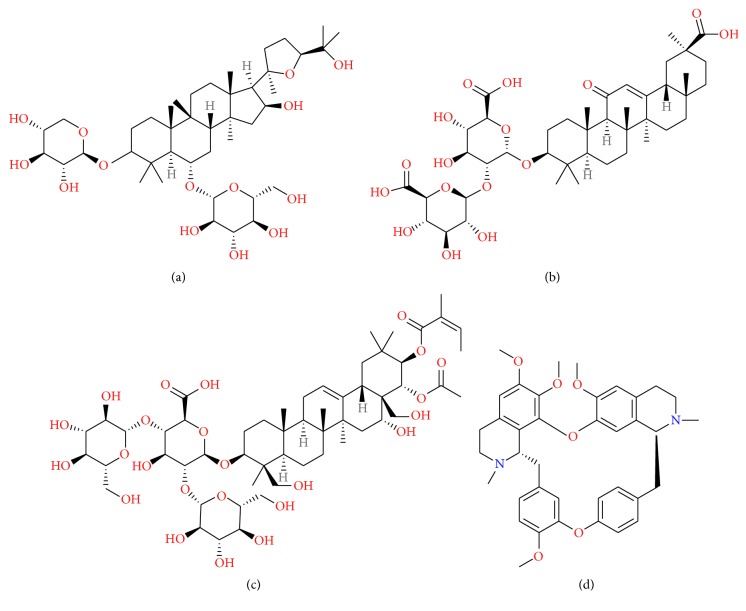
Structure of natural components: (a) Astragaloside IV, (b) Glycyrrhizin, (c) Beta-aescin, and (d) Tetrandrine.

Astragaloside IV (AsIV) (Figure 2(a)), the major active ingredient extracted from the Chinese herb* Astragalus membranaceus*, has been widely applied to treat cardiovascular disease [83]. Luo et al. have demonstrated that postischemic treatment of AsIV significantly reduced infarct volume in t-MCAO mice, resulting partly from its antioxidant properties [84]. Recent study showed that the protective effects on focal cerebral ischemia of AsIV might be linked with the antioxidation, downregulating the expressions of iNOS and upregulating nerve growth factor (NGF) in t-MCAO rats [85]. Moreover, AsIV has potential to inhibit cytokine-stimulated expression of E-selectin and VCAM-1 on the surface of HUVECs and leukocytes adhesion to endothelial cells in a dose- and time-dependent manner [18, 86]. The molecular weight (785 Da), hydrogen-bonding capacity (23), and NROTB (16) of AsIV all exceed the favorable value <500 Da, <12, and <10, respectively, which result in the extremely low level of AsIV in the brain compared with that of other tissues [17]. In the rats given AsIV at 3.0 mg/kg intravenously, half-life (*t*_1/2_) of the drug is 0.97 h, while first prominent enhancement of barrier permeability occurs at 3–6 hours after stroke. As a result, AsIV may not pass through BBB sufficiently to be delivered into the CNS and exert anti-inflammation on injured brain. Novel data revealed that AsIV was able to exert a neuroprotective effect and improve the outcome in short term, as a consequence of its inhibiting neutrophils adhesion and infiltration through attenuating upregulation of MAC-1 and ICAM-1, as well as suppressing proinflammatory factors (TNF-*α* and IL-1*β*) [86]. AsIV was also confirmed to attenuate TNF-*α*-induced upregulation of CAMs mRNA [87] and strongly inhibit neutrophil infiltration and activation [88]. In addition, AsIV could downregulate the level of ICAM-1 mRNA [89] and decrease the serum levels of TNF-*α*, MCP-1, and ICAM-1 [90].

Glycyrrhizin (GL) (Figure 2(b)), a triterpene present in the roots and rhizomes of Licorice, has been shown to have anti-inflammatory and antioxidative effects [91]. Due to large molecular weight (823 Da), high hydrogen-bonding capacity (24), and high molecular flexibility (NROTB: 12), pure GL administrated intravenously at 100 mg/kg to rats was not detected in the brain [17]. Another study also mentioned that GL demonstrated a negligible distribution in the brain [92]. Meanwhile, *t*_1/2_ of GL was determined 1.8 h at the dose of intravenously 200 mg/kg [93] and GL bound highly (97–99%) to rat or human serum albumin [94, 95], probably contributing to difficulty to cross the BBB [17]. Treatment with GL significantly reduced the levels of ICAM-1 in TNF-*α*-stimulated HaCaT cells and inhibited subsequent monocytes adhesion to keratinocytes [96]. Research showed that administration of GL significantly improved neurological outcomes via decreasing inflammatory cytokines expression (TNF-*α* and IL-1*β*) induced by NF-*κ*B and alleviating neutrophil infiltration in ischemic spinal cord [19]. In Japan, Stronger Neominophagen C (SNMC), of which GL is the major active ingredient, has been used to treat chronic hepatitis for more than 30 years. A recent study displayed that SNMC exerted neuroprotective effects through suppressing microglia activation and neutrophil infiltration in the ischemic brain in MCAO rats [91]. GL was potentially useful for inflammatory response by suppressing the adherence of neutrophils to the vascular endothelium [97–99], with evidence of reduced activity of myeloperoxidase (MPO) [100, 101].

Sodium *β*-aescin (Figure 2(c)), extracted from the seeds of Horse chestnut and divided into four types, has been widely used clinically as antiedema and antiexudation drug and all types are active ingredients. Sodium *β*-aescin significantly reduced cerebral infarct volume and water content and improved the neurological deficits through its antioxidant activity [102] with fewer side effects and complications [103]. Sodium *β*-aescin with large molecule weight and high hydrogen-bond capacity which are far more than the favorable values like AsIV is thought perhaps not to penetrate the BBB efficiently in principle, while there is no direct evidence to describe its tissue distribution, especially in the brain. Data showed that pretreatment with sodium *β*-aescin for 7 d could remarkably alleviate cerebral ischemia/reperfusion injury induced by MCAO, resulting from significantly reducing the cerebral infarct volume and ameliorating the neurological deficits through downregulating the protein expressions of ICAM-1 and E-selectin and inhibiting the migration of neutrophils after cerebral ischemia/reperfusion [20]. Several experiments also suggested that aescin treatment decreased neutrophil recruitment, adherence, and activation [104, 105], which could explain in part the potential benefit of the drug in the prevention of inflammatory response. Another data demonstrated that aescin influenced the later phases of inflammation (leukocyte migration) [106]. Likewise, although there is lack of evidence in brain distribution of Tetrandrine (TTD) (Figure 2(d)), a large-molecule natural chemical product purified from Fourstamen stefania root, it is certified to inhibit neutrophil recruitment by downregulating the expression of ICAM-1 and improve stroke outcomes [107]. Many data sets suggested that the protective effect of TTD against inflammation could be explained by significantly inhibiting neutrophil priming and activation, thereby abolishing subsequent infiltration [108, 109] via suppressing upregulation of MAC-1 [110–112].

**Figure 3:**
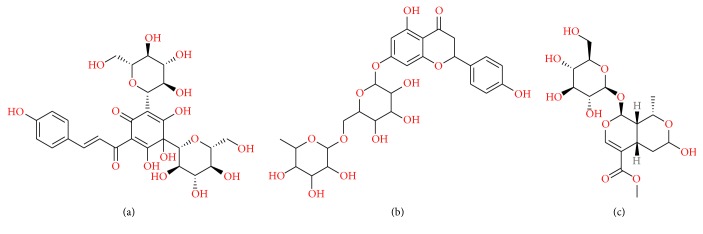
Structure of natural components: (a) Hydroxysafflor yellow A, (b) Naringin, and (c) Morroniside.

**Figure 4:**
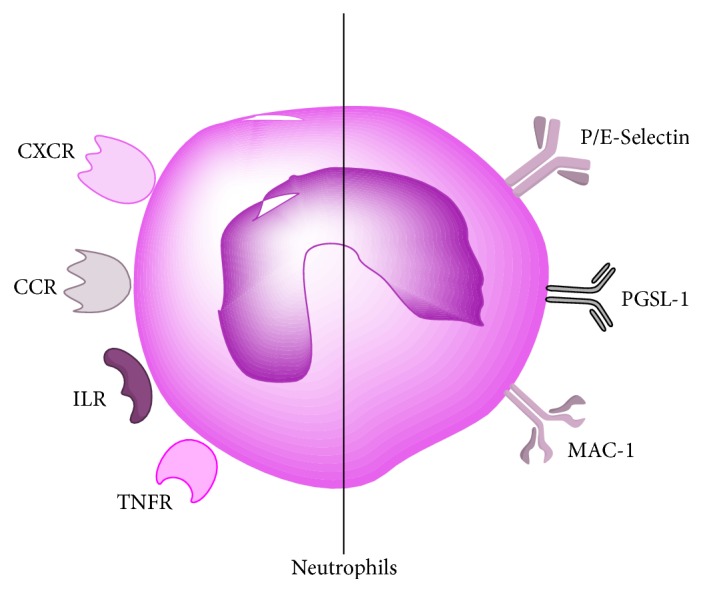
The receptors (the left) related to chemokines (CCR, CXCR) and cytokines (TNFR, ILR), and CAMs (the right) on neutrophils.

Hydroxysafflor yellow A (HSYA) (Figure 3(a)), an active ingredient of* Carthamus tinctorius* L. flowers, has been reported to treat cerebrovascular and cardiovascular diseases because of its multiple biological activities. Zhang et al. demonstrated that HSYA significantly reduced the expression of the proinflammatory mediators such as IL-1*β*, IL-4, TNF-*α*, and iNOS, as well as inhibiting Ab1-42-induced neuroinflammation* in vitro* [113]. After injected intravenously in mice, HSYA was found existing in plasma, kidney, liver, lung, heart, and spleen but not in the brain [114], resulting partly from its large molecule weight and high hydrogen-bonding capacity. It has also been observed that HSYA had potential to inhibit the elevation of TNF-*α*, IL-6, ICAM-1, and VCAM-1 induced by lipopolysaccharide (LPS) to protect endothelial cells against inflammatory injury [115]. HSYA was effective to protect LPS-induced high expression of endothelium adhesive molecule, such as ICAM-1 and E-selectin [116–118]. HSYA significantly reduced the cell surface level of TNFR1 and inhibited TNF-*α*-induced adhesion of macrophage to endothelium [119]. However, there is little evidence that HSYA exerts anti-inflammation on brain injury through directly inhibiting neutrophil infiltrating; thus, additional researches are required to confirm the postulation. Naringin, a major antioxidant constituent in* Citrus* fruits and herbs, is similar to HSYA regarding the absent evidence of directly inhibiting neutrophil infiltrating after cerebral ischemia (Figure 3(b)). Naringin has been used as an effective anti-inflammatory medicine. One study reported that the free forms of Naringin and Naringenin were not detected in all the tissues assayed, and Naringenin glucuronides were present in liver and kidney but not in spleen, brain, and heart [120]. Naringin was tested to inhibit upregulation levels of proinflammatory mediators (ICAM-1, MIP-2) and downregulation of anti-inflammatory mediators [121]. Treatment of Naringin (50 and 100 mg/kg) for seven days significantly improved locomotor activity and reduced resistance to lateral push and transfer latency in cerebral ischemia/reperfusion [122]. Naringin prevented cigarette smoke-induced infiltration of neutrophils and activation of MPO and matrix metalloproteinase-9 [123]. Naringin dose-dependently suppressed TNF-*α*-induced expressions of CAMs and chemokines at both the mRNA and protein levels, as well as the adhesion of monocytes to the TNF-*α*-stimulated HUVECs [124]. Also, Naringin reduced the stimuli-induced ICAM-1 expression [125, 126], contributing to the inhibition of monocytes adhesion to endothelial cells [126, 127].

Furthermore, Morroniside (Figure 3(c)) is an important constituent of the traditional Chinese medicine Fructus Corni with several bioactivities. Some findings demonstrated that Morroniside exerted neuroprotective effects on the brain damage induced by focal cerebral ischemia, related to its antioxidant and antiapoptotic properties in the brain [128]. Pharmacokinetic study revealed that Morroniside administrated intravenously and orally was absorbed and eliminated rapidly in rats and absolute bioavailability of Morroniside was lower, and no Morroniside was found in brain [129]. Morroniside promoted angiogenesis, in part through accelerating endothelial progenitor cells (EPCs) proliferation in ischemic stroke models [130]. EPCs derived from bone marrow mobilize into the blood and home to the site of ischemic region through adherence, differentiation, proliferation, and migration to participate in tissue vascularization [131]. Processes of EPCs mobilizing from bone marrow to ischemic area partly occur in the peripheral blood. In addition, insulin-like growth factor-1 (IGF-1) and Erythropoietin (EPO) are generated by peripheral organs like liver and kidney, respectively, and delivered into the brain through cerebrovasculature to participate in angiogenesis, which can be modulated peripherally by medicine that cannot penetrate the BBB [132, 133].

## 5. Conclusion

Although majority of agents showed anti-inflammation activity in cerebral parenchyma after stroke, we cannot ignore the problem that they were at very low concentrations or not detected in the brain tissue. Therefore, in the coagulation models, they may play an anti-inflammatory role due to their inhibiting neutrophil infiltration in peripheral circulation system. And such pathway was attributed to poor membrane permeability decided by physicochemical properties of agents and slight BBB leakage depending on type of stroke models. Additionally, administration time, frequency, and method of drugs influence the concentration in the brain. In summary, if drugs could achieve effective concentration in the cerebral parenchyma, they can function centrally and peripherally; otherwise, the latter may be dominating. Also, Kim et al. have reported that neutrophil counts were associated positively with the severity of stroke, while lower lymphocyte counts were linked with poor functional outcome [134]. When inhibiting neutrophils at the level of inflammatory mediators, will these drugs cripple the number and function of lymphocytes? There is no clear answer. Thus, much more work are required to do to explore inflammation process, the anti-inflammatory mechanisms about medicine localized in the brain at a very low concentration, and pharmacokinetics of drugs in various stroke models, as well as difference of BBB disruption in all types of models at a molecule level. Also, can different therapies be given as the infarct types of patients and time course of stroke progression?

## Figures and Tables

**Table 1:** Major cell adhesion molecules (CAMs) involved in neutrophils infiltration.

Categories	Cell adhesion molecules	Expressed on
Selectins	P-Selectin	Endothelium, platelet
	E-Selectin	Endothelium, leukocyte
	L-Selectin	Endothelium, leukocyte

Integrins	CD11b/CD18 (MAC-1)	Leukocyte
	CD41	Platelet

Immunoglobulin superfamily	ICAM-1	Endothelium, leukocyte
	ICAM-2	Endothelium, platelet
	VCAM-1	Endothelium

Mucin-like family	PSGL-1	Leukocyte
